# Electronic healthcare records and external outcome data for hospitalized patients with heart failure

**DOI:** 10.1038/s41597-021-00835-9

**Published:** 2021-02-05

**Authors:** Zhongheng Zhang, Linghong Cao, Rangui Chen, Yan Zhao, Lukai Lv, Ziyin Xu, Ping Xu

**Affiliations:** 1 Department of Emergency Medicine, Sir Run Run Shaw Hospital, Zhejiang University School of Medicine, Hangzhou, 310016 Zhejiang China; 2 Key Laboratory of Emergency and Trauma, Ministry of Education, College of Emergency and Trauma, Hainan Medical University, Haikou, 571199 China; 3 Emergency Department, Zigong Fourth People’s Hospital, 19 Tanmulin Road, Zigong, Sichuan China; 4 Artificial Intelligence Key Laboratory of Sichuan Province, Zigong, 643000 China; 5 Medical Big Data and Artificial Intelligence Laboratory of Zigong Fourth People’s Hospital, Zigong, 643000 China

**Keywords:** Heart failure, Epidemiology

## Abstract

Heart failure is one of the most important reasons for hospitalization among elderly individuals and is associated with significant mortality and morbidity. Epidemiological studies require the establishment of high-quality databases. Several datasets that primarily involve heart failure populations have been established in Western countries and have generated many high-quality studies. However, no such dataset is available from China. Due to differences in genetic background and healthcare systems between China and Western countries, the establishment of a heart failure database for the Chinese population is urgently needed. We performed a retrospective single-center observational study to collect data regarding the characteristics of heart failure patients in China by integrating electronic healthcare records and follow-up outcome data. The study collected information for a total of 2,008 patients with heart failure, containing 166 attributes.

## Abstract (summary)

**Table:** 

Measurement(s)	Clinical Data • Demographic Data • Comorbidity • drug administration • Follow-Up Information • heart failure
Technology Type(s)	Electronic Medical Record
Factor Type(s)	destination after hospital discharge
Sample Characteristic - Organism	Homo sapiens
Sample Characteristic - Location	China

Machine-accessible metadata file describing the reported data: 10.6084/m9.figshare.13064054

## Background & Summary

Heart failure (HF) affects over 6 million people in the United States, with an estimated incidence of 21 per 1000 people in the elderly people^1^. By using mathematical prediction models, heart failure is estimated to affect 8 million people over the age of 18^2^. Heart failure is one of the most important reasons for hospitalization among elderly individuals and is also associated with significant mortality and morbidity. It has been reported that the mortality ranges from 20% to 60% one year after hospitalization for acute HF^3–6^, depending on comorbidities and coexisting medical conditions. Many cohort studies have been carried out for epidemiological investigations of hospitalized patients with HF. For instance, the Cleveland Heart Disease dataset, which contains 75 variables for 303 patients, is mainly used for practising machine learning algorithms^7^. The Nationwide Inpatient Sample (NIS) is a publicly available database from the Healthcare Utilization Project (HCUP) that is supported by the Agency for Healthcare Research and Quality. This dataset contains many patients with heart diseases, but the variables/attributes included in this dataset are not specifically designed for HF^8–10^. The Medical Information Mart for Intensive Care (MIMIC) database contains data associated with >60,000 distinct hospital admissions to critical care units between 2001 and 2012. Many of the patients have a HF diagnosis, and thus MIMIC is a good resource for testing research hypotheses related to critically ill HF patients^11^. However, these studies are either designed with attributes that are limited in number or not specific for HF. In other words, data collection was dictated by expert knowledge, and only variables deemed important were entered into the data collection form. The determination of feature variable inclusion/exclusion is largely driven by expertise and previous studies. Such a dataset can be used only to address a limited number of clinical questions. For example, the ESC-Heart Failure Association (HFA) EURObservational Research Programme (EORP) generated a large dataset that contained specifically HF patients, and a large amount of data that are routinely collected during clinical practice were abandoned. To the best of our knowledge, this is the largest HF dataset in the world, including 337 cardiology centres from 33 ESC Member countries^12^. In essence, many trivial attributes may work together to influence the clinical outcome. Thus, a dataset including all aspects of individual patient-level data can help disentangle complex relationships among attributes. In the era of big data, the electronic healthcare records are able to produce a large amount of data related to a given HF patient. These multiparameter relational databases may or may not be related to a given research question. Different studies and analyses require different variables. Making such a publicly available dataset can help to encourage data reuse, thereby promoting more medical knowledge discovery.

Our study aimed to establish a HF database based on electronic healthcare records. Data on subsequent hospital admissions and mortality were obtained at mandatory follow‐up visits at 28 days, 3 months and 6 months (if the patient was unable to reach the clinical centre, the follow‐up visit was replaced by a telephone call). The study was a retrospective study enrolling hospitalized patients with heart failure from December 2016 to June 2019. Patients were enrolled from Zigong Fourth People’s Hospital. Data were extracted from electronic healthcare records. However, this is a single-centre dataset, covering only Chinese patients. Findings with these data alone may not have convincing generalizability. Researchers may combine this dataset with other heart failure cohort data for a larger-scale study.

## Methods

### Study setting and population

The study was conducted at Zigong Fourth People’s Hospital, Sichuan, China from December 2016 to June 2019, and was approved by the ethics committee of Zigong Fourth People’s Hospital (Approval Number: 2020-010). Informed consent was waived due to the retrospective design of the study. The study complies with the Declaration of Helsinki.

Electronic healthcare records of consecutive patients with a diagnosis of HF were reviewed. We included all types of heart failure including acute HF, chronic HF, left HF, right HF, or a mixture of all. Heart failure was defined according to the European Society of Cardiology (ESC) criteria^13^:

1. The presence of symptoms and/or signs of HF. Typical symptoms include breathlessness, orthopnoea, paroxymal nocturnal dyspnea, reduced exercise tolerance, fatigue, tiredness, increased time to recover after exercise and ankle swelling. Typical signs include elevated jugular venous pressure, hepatojugular reflux, third heart sound (gallop rhythm) and laterally displaced apical impulse.
2. Elevated levels of BNPs (BNP >35 pg/mL and/or NT‐proBNP >125 pg/mL)
3. Objective evidence of other cardiac functional and structural alterations underlying HF.
4. In case of uncertainty, a stress test or invasively measured elevated LV filling pressure may be needed to confirm the diagnosis.

Patients who had a diagnosis of heart failure on hospital admission were enrolled in our study. The diagnosis was recorded with ICD- 9 in the EHR (Table 1).

**Table 1:** ICD-9 code for the diagnosis of heart failure.

Code	Description
428	Heart failure
4280	Congestive heart failure, unspecified
4281	Left heart failure
4282	Systolic heart failure
42820	Systolic heart failure, unspecified
42821	Acute systolic heart failure
42822	Chronic systolic heart failure
42823	Acute on chronic systolic heart failure
4283	Diastolic heart failure
42830	Diastolic heart failure, unspecified
42831	Acute diastolic heart failure
42832	Chronic diastolic heart failure
42833	Acute on chronic diastolic heart failure
4284	Combined systolic and diastolic heart failure
42840	Combined systolic and diastolic heart failure, unspecified
42841	Acute combined systolic and diastolic heart failure
42842	Chronic combined systolic and diastolic heart failure
42843	Acute on chronic combined systolic and diastolic heart failure
4289	Heart failure, unspecified

### Variables and attributes

Data collected for the dataset included three broad categories: demographic data, baseline clinical characteristics, comorbidities, laboratory findings, drugs and outcomes. Demographic data were entered manually into the EMR system by the nurses on admission if a patient first visited our hospital. Otherwise, demographic data could be automatically extracted from previous visits. Some missing or error data were checked if they were identified by the nurses. To ensure the accuracy and consistency of data entry, a drop-down list was used for some variables in our EMR system, such as sex, department of admission and occupation. Laboratory tests and drugs were electronically entered by physicians and/or lab workers. Data in the EMR were extracted by SQL query to establish the current database. The accuracy of the SQL query was then checked manually by randomly selecting 50 patients. Many data items were recorded in Chinese in the electronic healthcare record database, thus the largest challenge is the language barrier. All the lab test items, examinations, drug names and diagnoses were recorded in Chinese in the electronic healthcare record database. To address this problem, all Chinese terms were translated to English by the principal investigators (Z.Z., P.X. and L.C.).

The demographic data were obtained from the first sheet of the medical records and included age, sex, height, body weight, admission ward, type of admission (emergency vs. nonemergency), occupation, discharge department, admission date, visit times, and marital status.

Baseline clinical characteristics were measured on the day of hospital admission and included body temperature, pulse, respiration rate, systolic blood pressure, diastolic blood pressure, mean arterial blood pressure, weight, height, body mass index (BMI), type of heart failure, New York Heart Association (NYHA) cardiac function, Killip Grade (Class 1 No rales, no 3rd heart sound; Class 2 Rales in <1⁄2 lung field or presence of a 3rd heart sound; Class 3 Rales in >1⁄2 lung field–pulmonary oedema; Class 4 Cardiogenic shock–determined clinically), and Glasgow Coma Scale (GCS) score. Echocardiographic findings included left ventricular ejection fraction (LVEF), left ventricular end diastolic diameter, mitral valve peak E wave velocity (m/s), mitral valve peak A wave velocity (m/s), E/A, tricuspid valve regurgitation velocity, and tricuspid valve regurgitation pressure.

Comorbidities included a medical history of myocardial infarction, congestive heart failure, peripheral vascular disease, cerebrovascular disease, dementia, chronic obstructive pulmonary disease (COPD), connective tissue disease, peptic ulcer disease, diabetes, moderate-to-severe chronic kidney disease, hemiplegia, leukaemia, malignant lymphoma, solid tumour, liver disease and AIDS. The Charlson Comorbidity Index (CCI) was calculated by summing all comorbidity points described above^14^. A minority of patients were not coded as having a diagnosis of “congestive heart failure” in the comorbidity list because they did not have a past history of congestive heart failure on admission. They were diagnosed with HF for the first time in the index hospitalization. The comorbidities were taken from the admission notes.

Laboratory findings were obtained from day one of hospital admission, including serum creatinine, urea, uric acid, glomerular filtration rate, cystatin, white blood cell count, monocyte ratio, monocyte count, red blood cell count, coefficient of variation of red blood cell distribution width, standard deviation of red blood cell distribution width, mean corpuscular volume, haematocrit, lymphocyte count, mean haemoglobin volume, mean haemoglobin concentration, mean platelet volume, basophil ratio, basophil count, eosinophil ratio, eosinophil count, haemoglobin, platelet, platelet distribution width, platelet haematocrit, neutrophil ratio, neutrophil count, D-dimer, international normalized ratio, activated partial thromboplastin time, thrombin time, prothrombin activity, prothrombin time ratio, fibrinogen, high sensitivity troponin, myoglobin, carbon dioxide binding capacity, calcium, potassium, chloride, sodium, inorganic phosphorus, serum magnesium, creatine kinase isoenzyme to creatine kinase, hydroxybutyrate dehydrogenase to lactate dehydrogenase, hydroxybutyrate dehydrogenase, glutamic oxaloacetic transaminase, creatine kinase, creatine kinase isoenzyme, lactate dehydrogenase, brain natriuretic peptide, high sensitivity protein, nucleotidase, fucosidase, albumin, albumin/globulin ratio, cholinesterase, glutamyltranspeptidase, glutamic pyruvic transaminase, glutamic oxaliplatin, indirect bilirubin, alkaline phosphatase, globulin, direct bilirubin, total bilirubin, total bile acid, total protein, erythrocyte sedimentation rate, cholesterol, low-density lipoprotein cholesterol, triglyceride, high-density lipoprotein cholesterol, homocysteine, apolipoprotein A, apolipoprotein B, lipoprotein, pH, standard residual base, standard bicarbonate, partial pressure of carbon dioxide, total carbon dioxide, methemoglobin, haematocrit blood gas, reduced haemoglobin, potassium ion, chloride ion, sodium ion, glucose blood gas, lactate, measured residual base, measured bicarbonate, carboxyhemoglobin, body temperature blood gas, oxygen saturation, partial oxygen pressure, oxyhemoglobin, anion gap, free calcium, and total haemoglobin.

Primary drug categories included in our dataset were diuretics, inotropes, and vasodilators. The diuretics included furosemide, torasemide and spironolactone. Inotropes included deslanoside, dobutamine, digoxin, isoprenaline and milrinone. Vasodilators included isosorbide mononitrate and nitroglycerin.

Outcome variables included discharge date of the index hospital, vital status at hospital discharge, death within 28 days, readmission within 28 days, death within 3 months, readmission within 3 months, death within 6 months, readmission within 6 months, time to death (days from index hospital admission), time to readmission (days from index hospital admission), return to emergency department within 6 months, and time to visit emergency department within 6 months. The variable “*DestinationDischarge*” was recorded after hospital discharge, and the variable “*outcome.during.hospitalization*” was recorded after the decision to discharge was made.

## Data Records

The study generated a single dataset, that contained information on 166 attributes of 2008 hospitalized patients from December 2016 to June 2019. The dataset is available at PhysioNet (10.13026/8a9e-w734)^15^. Missing values are indicated with blanks. Detailed information on variable specifications is included in a variable description file.

## Technical Validation

The present study was a retrospective design. Information on eligible patients was collected at Zigong Fourth People’s Hospital. First, the required data were exported from the electronic healthcare database with the assistance of the information technology technician. The exported data were then checked by expert emergency and critical care physicians; if outliers in each variable and contradictions within data were detected, data were validated by another investigator. The outliers and contradictions were judged by expert emergency and critical care physicians. Data on subsequent hospital admissions and mortality were obtained at mandatory follow‐up visit at 28 days, 3 months and 6 months (if the patient was unable to reach the clinical centre, the follow‐up visit was replaced by a telephone call).

Data were finalized and fully anonymized on June 8, 2020.

### Baseline characteristics of included patients

**Fig. 1:**
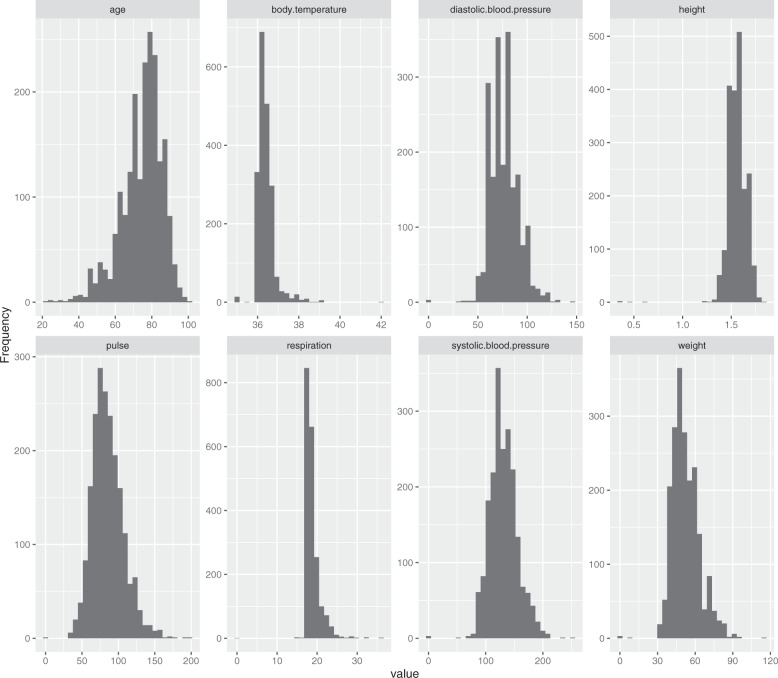
Histogram showing the distribution of numeric attributes at baseline.

**Fig. 2:**
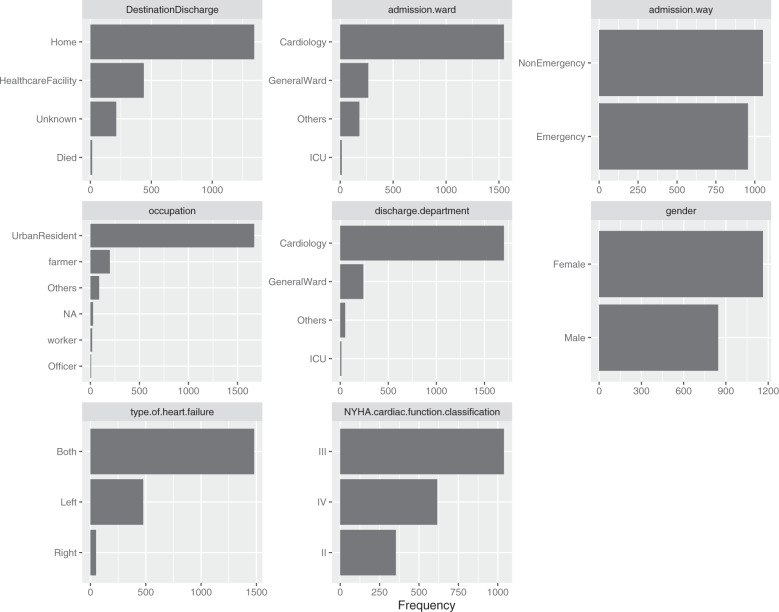
Bar chart showing the distribution of discrete attributes at baseline.

The overall mortality rate at hospital discharge was 1% (14/2008). A total of 212 patients were discharged to unknown places (212/2008, 11%), 1344 patients were discharged home (67%) and 438 patients were discharged to healthcare facilities (22%). Most patients were admitted to the department of cardiology (1547/2008, 77%), followed by the general ward (265/2008, 13%), others (181/2008, 9%) and the ICU (15/2008, 1%). There was also a significant difference between emergency and nonemergency patients (Online-only Table 1). The distributions of the baseline characteristics are shown in Fig. 1, Fig. 2 and Online-only Table 1.

## Online-only Table

**Online-only Table 1:** Baseline characteristics of included heart failure patients.

Variables	Total (n = 2008)	Emergency (n = 956)	Non-Emergency (n = 1052)	p
Destination of discharge, n (%)				<0.001
Died	14 (1)	10 (1)	4 (0)	
Unknown	212 (11)	125 (13)	87 (8)	
HealthcareFacility	438 (22)	261 (27)	177 (17)	
Home	1344 (67)	560 (59)	784 (75)	
admission.ward, n (%)				<0.001
Cardiology	1547 (77)	688 (72)	859 (82)	
GeneralWard	265 (13)	195 (20)	70 (7)	
ICU	15 (1)	13 (1)	2 (0)	
Others	181 (9)	60 (6)	121 (12)	
admission.way, n (%)				<0.001
Emergency	956 (48)	956 (100)	0 (0)	
NonEmergency	1052 (52)	0 (0)	1052 (100)	
occupation, n (%)				0.182
farmer	198 (10)	97 (10)	101 (10)	
Officer	7 (0)	1 (0)	6 (1)	
Others	89 (4)	49 (5)	40 (4)	
UrbanResident	1670 (84)	789 (84)	881 (85)	
worker	17 (1)	6 (1)	11 (1)	
discharge.department, n (%)				<0.001
Cardiology	1703 (85)	763 (80)	940 (89)	
GeneralWard	241 (12)	174 (18)	67 (6)	
ICU	12 (1)	7 (1)	5 (0)	
Others	52 (3)	12 (1)	40 (4)	
visit.times, n (%)				<0.001
1	1860 (93)	910 (95)	950 (90)	
2	120 (6)	40 (4)	80 (8)	
3	20 (1)	5 (1)	15 (1)	
4	6 (0)	1 (0)	5 (0)	
5	2 (0)	0 (0)	2 (0)	
marital.status, n (%)				<0.001
Divorced	30 (2)	23 (2)	7 (1)	
Married	1304 (66)	599 (64)	705 (68)	
Others	108 (5)	24 (3)	84 (8)	
Unmarried	9 (0)	5 (1)	4 (0)	
Widowed	529 (27)	290 (31)	239 (23)	
gender, n (%)				0.664
Female	1163 (58)	559 (58)	604 (57)	
Male	845 (42)	397 (42)	448 (43)	
age, Median (IQR)	77 (68, 83)	78 (69, 83)	76 (68, 82)	0.001
body.temperature, Median (IQR)	36.3 (36.2, 36.5)	36.3 (36.2, 36.5)	36.3 (36.2, 36.5)	0.839
pulse, Median (IQR)	82 (70, 98)	85 (72, 100)	80 (69, 96)	<0.001
respiration, Median (IQR)	19 (18, 19)	19 (18, 20)	19 (18, 19)	<0.001
systolic.blood.pressure, Median (IQR)	130 (113, 146.25)	130 (114, 150)	128 (112, 144.25)	0.005
diastolic.blood.pressure, Median (IQR)	76 (65, 85)	78 (66, 88)	76 (65, 84)	0.006
map, Median (IQR)	93.33 (83.33, 104.67)	95.33 (83.33, 106.67)	93.33 (83.33, 103.33)	0.002
weight, Median (IQR)	50 (45, 60)	50 (45, 60)	50 (45, 60)	0.664
height, Median (IQR)	1.56 (1.5, 1.62)	1.56 (1.5, 1.62)	1.56 (1.5, 1.62)	0.683
BMI, Median (IQR)	20.76 (18.49, 23.44)	20.76 (18.42, 23.44)	20.76 (18.49, 23.46)	0.744
type.of.heart.failure, n (%)				0.051
Both	1480 (74)	684 (72)	796 (76)	
Left	477 (24)	250 (26)	227 (22)	
Right	51 (3)	22 (2)	29 (3)	
NYHA.cardiac.function.classification, n (%)				0.535
II	353 (18)	162 (17)	191 (18)	
III	1039 (52)	490 (51)	549 (52)	
IV	616 (31)	304 (32)	312 (30)	
Killip.grade, n (%)				<0.001
I	527 (26)	207 (22)	320 (30)	
II	1029 (51)	505 (53)	524 (50)	
III	392 (20)	213 (22)	179 (17)	
IV	60 (3)	31 (3)	29 (3)	
